# Gender Differences in Self-Reported Symptoms of Depression among Patients with Acute Coronary Syndrome

**DOI:** 10.1155/2012/109251

**Published:** 2012-03-26

**Authors:** Lorraine Frazier, Erica Yu, Jennifer Sanner, Fang Liu, Malini Udtha, Stanley Cron, Stephanie Coulter, Roberta C. Bogaev

**Affiliations:** ^1^School of Nursing, University of Texas Health Science Center at Houston, 6901 Bertner Avenue 577, Houston, TX 77030, USA; ^2^Texas Heart Institute, St. Luke's Episcopal Hospital, 6624 Fannin, Suite 2780, Houston, TX 77030, USA

## Abstract

This study examined the prevalence of self-reported depressive symptoms and the self reported somatic depressive symptoms as measured by the Beck Depression Inventory-II (BDI-II) among patients hospitalized for acute coronary syndrome (ACS), and explored the impact of gender on both. A convenience sample of 789 adults (248 women and 541 men) was recruited for the study during hospital admission for ACS and participants were screened for self-reported depressive symptoms. BDI-II scores of ≥14 indicate a moderate level of depressive symptoms and this cut-off score was used to categorize patients into depressed and non-depressed groups. Pearson chi-square tests for independence (categorical variables) and *t* tests for independent samples (continuous variables) were used for gender comparisons. Results showed that depressive symptoms during ACS episodes were different between women and men. Women reported greater overall depressive symptoms (BDI-II mean = 11.89, S.D. = 9.68) than men (BDI-II mean = 9.00, S.D. = 7.93) (*P* < 0.000). Significantly more women (7.66%) were identified positive for somatic depressive symptoms (sleep and appetite disturbances and fatigue) than men (2.22%) (*P* = 0.0003). Findings support that there are gender differences in depressive symptoms experienced by patients hospitalized for ACS. Somatic symptoms of depression may be important indicators of depression especially among female ACS patients.

## 1. Introduction

Each year 2.4 million Americans are hospitalized for acute coronary syndrome (ACS) [1]. Growing evidence supports that post-ACS depression is an independent risk factor for future cardiac events and mortality [2–4], and females are disproportionately affected [5]. An estimated 7.5 million females live with a history of ACS [1] and up to 47% of females report symptoms of depression when screened for depression during hospitalization for ACS [6]. Depression is the most common mental illness in females and females have twice the risk of major depressive disorder (MDD) as compared with males [7, 8]. Data from National Comorbidity Survey (NCS) supports the gender difference life time risk for MDD (21% in females and 13% in males) [9]. Population studies from Canada [10], Germany [11], and Switzerland [12] all report that females are at least twice as likely as males to suffer from MDD. In addition to the gender difference in prevalence of depression episodes, the depression symptoms that differ between males and females are heterogeneous with complex clusters of depressive symptoms [13, 14].

Data from large epidemiological studies reveals that males and females often report distinct differences in the self-reported somatic depressive symptoms of depression [15–17]. Somatic depressive symptoms include sleep disturbance, appetite disturbance, and fatigue for at least two weeks [15–17]. Silverstein interviewed college students and found that female students had higher somatic depressive symptoms than male students [18]. Self-reported data from NCS also indicated that females reported twice the frequency of somatic depression symptoms as compared to males [19]. Although higher rates of depression have been reported in patients with ACS, no known studies have specifically evaluated gender differences in specific depressive symptoms reported during the ACS event. The purpose of this study was to examine the gender differences and prevalence of self-reported depressive symptoms and self-reported somatic depressive symptoms, as measured by the Beck Depression Inventory II (BDI-II) [20], in patients hospitalized for ACS.

## 2. Methods

This was a cross-sectional observational study with a convenience sample of 789 adults (248 females and 541 males) hospitalized for ACS. The study included all participants meeting the inclusion criteria of a larger study looking at the interactions of genetics and depression on ACS outcomes. The overall rate for males verses females was consistent with the gender ratio for ACS admitted to the facility. Participants were recruited during hospital admission for ACS and were screened for self-reported depressive symptoms, by trained research nurses, using the BDI-II. Depression was defined as a BDI-II score of ≥14 indicating at least moderate symptoms of depression [20].

The study was approved by the University's Committee for the Protection of Human Subjects Internal Review Board. After informed consent was obtained, by trained research nurses, the BDI-II was administered when the patients were stable enough during their hospital stay to answer the questions (1–3 days after hospital admission). Inclusion criteria consisted of a confirmed diagnosis of ACS and 18 years of age or older. ACS was defined as chest pain or symptoms suggestive of ACS lasting for more than 15 minutes with new transient or persistent ST-wave ischemic electrocardiogram (ECG) changes and ultimate disposition as (1) acute myocardial infarction (AMI), including ST-wave elevation infarction and non-ST wave elevation infarction or (2) unstable angina [19]. The final ACS diagnosis was verified through their hospital inpatient medical records.

The BDI-II is a reliable and well-validated 21-item depression screening scale that uses a forced-choice 4-alternative response format that has been used in the ACS population [20]. The BDI-II has been used for 35 years to identify and assess depressive symptoms and reports high reliability (.80) regardless of the population [21, 22]. The construct validity of BDI-II has been established and has shown to be able to differentiate between depressed and non-depressed individuals. Higher coefficient alphas (.92 for outpatients and  .93 for college students) have been reported for the BDI-II when compared to the previous version of the Beck Depression Inventory (BDI) [22]. Test-retest reliability was studied with baseline data obtained on 26 outpatients and therapy sessions one week apart (correlation  .93, *P* < 0.001) [22]. The internal consistency of the BDI-II in patients with ACS has an alpha value of 0.87 [23]. Higher BDI-II scores indicate a greater number of depressive symptoms. BDI-II scores of ≥14 indicate a moderate level of depressive symptoms [20, 24]. This cut-off score has successfully been used to screen patients hospitalized for ACS (odds ratio, 7.82; 95% CI, 2.42 to 25.26; *P* = 0.0002) and the internal consistency of the BDI-II in patients with ACS has an alpha value of 0.87 [24]. 

Somatic depressive symptoms were measured using BDI-II question 16 (sleep disturbance), question 18 (appetite disturbance), and question 20 (fatigue). A patient was identified as positive for somatic depressive symptoms when the patient rated all three items as 1 or above. If a patient rated 0 on any of the three items, he or she was categorized as negative for somatic depressive symptoms. Pearson chi-square tests for independence (categorical variables) or *t*-tests for independent samples (continuous variables) were used to examine differences in demographic characteristics between males and females. Comparisons of depression ordinal scores and symptoms by gender were conducted with Wilcoxon rank sum and Pearson chi-square tests for independence, respectively. Degrees of freedom for the Pearson chi-square test were defined as follows: (*r* − 1) × (*c* − 1), where *r* = the number of rows and *c* = the number of columns. Significance level was set at *P* < 0.05. Data were analyzed using SAS 9.2 statistical software.

## 3. Results

### 3.1. Baseline Characteristics

 The sample consisted of 789 adult (248 females and 541 males) patients admitted with ACS (Table 1). Ages ranged from 28 to 96 years. A total of 88 (35%) females and 120 (22%) males had BDI-II scores of 14 or higher. Sixty percent of the patients were hospitalized for their first ACS event. The majority of the patients were Caucasian males (64%), with a mean age of 61.35 years (S.D. = 11.56). A significantly higher percentage of females were not married (51% for females and 27% for males, *P* < 0.0001), lived alone (25.5% for females and 17.6% for males, *P* = 0.0098), and were less physically active (35% for females and 24% for males, *P* = 0.0002) than males. More than half of females in the study had less than high school education (52%, *P* < 0.0001), while the majority of males had at least some college or college degree (60%). Females were more likely to currently be taking antidepressants (21.3%) than males (10.57%) (*P* < 0.0001) and significantly more females (31.7%) than males (16.3%) (*P* < 0.0001) reported a history of depression diagnosis or treatment.

### 3.2. Prevalence of Self-Reported Depressive Symptoms

Results showed that depression during ACS episodes are different between males and females. Significantly more females (43.5%) reported feeling depressed over the past year than compared to males (27.2%). During admission for ACS, the average BDI-II scores were significantly higher for females (mean = 11.89, S.D. = 9.68) than that for males (mean = 9.00, S.D. = 7.93) (*P* < 0.0001) indicating that the females reported greater depressive symptoms. Females reported significantly more sadness, self dislike, crying, indecisiveness, sleep disturbance, appetite disturbance, and loss of interest in sex than males (Table 2). Significantly more females (7.66%) were identified as positive for somatic depression symptoms (reported 1 or higher scores on all three questions) than males (2.22%) (*P* = 0.0003).

## 4. Discussion

The prevalence of depression in this study was significantly higher in the females (35%) than males (22%) (BDI-II scores ≥ 14). Of the 21 depressive symptoms evaluated by the BDI-II, 19 depressive symptoms were reported more frequently by females; 7 out of the 19 symptoms reaching statistical significance (*P* < 0.05). Although our results support the increase in somatic symptoms in females (sleep and appetite disturbances), as reported by Wenzel [15], self-reported symptoms of fatigue were not statistically different between males and females. 

Several methodological caveats must be considered when interpreting the data. The sample was a convenient sample largely made up of Caucasians with a high number of males, and participants were recruited from two large tertiary medical centers with high levels of acuity. Although similar patterns of somatic depressive symptoms have been reported from several epidemiological studies [15–17], other comorbid psychiatric, medical conditions, and treatments that may affect somatic symptoms should be considered. Some of the self-reported somatic symptoms may have been caused by the current ACS event. Despite these limitations, results from this study raise the possibility that females may have heterogeneous symptomology with various depression subtypes that may affect their morbidity and mortality after ACS events.

## 5. Conclusion

In conclusion, results from the present study support the hypothesis that there are gender differences in depression prevalence and self-reported somatic depressive symptoms, in patients hospitalized for ACS. Somatic symptoms may be important indicators of depression among ACS patients, especially female patients. The ability to diagnose and treat depression in these patients is an important intervention that would decrease subsequent ACS events. Future studies are needed to identify the biological and psychological mechanisms to explain this gender difference.

## Figures and Tables

**Table 1:** Comparison of demographic and clinical health characteristics of acute coronary syndrome patients.

Variables	(*n* = 789)	Women (*n* = 248)	Men (*n* = 541)	*P* value
Age at consent				0.0013*
M (year) ± SD	62.28 ± 12.03	64.31 ± 12.79	61.35 ± 11.56	
Range	28–96	29–96	32–95	
BMI				0.2995
M (lb/inch^2^) ± SD	30.13 ± 6.44	30.53 ± 7.91	29.95 ± 5.64	
Range	16.47–58.57	16.47–61.33	18.09–58.57	
BDI Score (0–63)				<0.0001*
M ± SD	9.93 ± 8.63	11.98 ± 9.68	9.00 ± 7.93	
Range	0–48	0–48	0–43	
Race				0.0274*
White	485 (61.47)	141 (56.85)	344 (63.59)	
Black	152 (19.26)	63 (25.40)	89 (16.45)	
Spanish/Hispanic Latino Descent	123 (15.59)	37 (14.92)	86 (15.90)	
Some other race	29 (3.68)	7 (2.82)	22 (4.07)	
Marital Status				<0.0001*
Single, Divorced, Separated, Widowed	272 (34.56)	126 (51.01)	146 (27.04)	
Married	515 (65.44)	121 (48.99)	394 (72.96)	
Living arrangement				0.0098*
Live with spouse or family member	630 (79.95)	184 (74.49)	446 (82.44)	
Live alone	158 (20.05)	63 (25.51)	95 (17.56)	
Education				<0.0001*
≤High school	340 (43.26)	129 (52.23)	211 (39.15)	
Some college/vocational Technical	217 (27.61)	74 (29.96)	143 (26.53)	
College degree and above	229 (29.13)	44 (17.81)	185 (34.32)	
Smoking				0.0054*
Yes, currently	178 (22.62)	54 (21.77)	124 (23.01)	
Yes, but quit (≤1 year)	290 (36.85)	74 (29.84)	216 (40.07)	
Never	319 (40.53)	120 (48.39)	199 (36.92)	
Alcohol				<0.0001*
Yes, currently	364 (46.43)	87 (35.22)	277 (51.58)	
Yes, but quit (≤1 year)	87 (11.10)	14 (5.67)	73 (13.59)	
Never	333 (42.47)	146 (59.11)	187 (34.82)	
Activity				0.0002*
More active	354 (45.10)	86 (34.82)	268 (49.81)	
Less active	218 (27.77)	88 (35.63)	130 (24.16)	
About same	213 (27.13)	73 (29.55)	140 (26.02)	
Depressed (BDI ≥14)	208 (26.36)	88 (35.48)	120 (22.18)	0.0001*
Depression questions				
Current antidepressant use	108 (13.95)	52 (21.31)	56 (10.57)	<0.0001*
History of Depression feeling	251 (32.39)	107 (43.50)	144 (27.22)	<0.0001*
History of depression diagnosis or treatment	164 (21.19)	78 (31.71)	86 (16.29)	<0.0001*

Note: values in parenthesis are percentages.

*Statistical significant at level of *P* < 0.05.

**Table 2:** Proportional comparison of each individual BDI-II question.

BDI-II 21 questions symptom present ≥1	All (*n* = 789)	Females (*n* = 248)	Males (*n* = 541)	*P* value
Q1:Sadness	206 (26.14)	91 (36.69)	115 (21.30)	<0.0001*
Q2:Pessimism	245 (31.05)	86 (34.68)	159 (29.39)	0.1362
Q3:Past failure	174 (22.08)	65 (26.21)	109 (20.19)	0.0583
Q4:Loss pleasure	330 (41.83)	103 (41.53)	227 (41.96)	0.9101
Q5:Guilty feelings	217 (27.57)	79 (31.98)	138 (25.56)	0.0611
Q6:Punish feelings	95 (12.09)	33 (13.36)	62 (11.50)	0.4583
Q7:Self dislike	149 (18.93)	59 (23.98)	90 (16.64)	0.0147*
Q8:Self criticalness	235 (29.90)	72 (29.15)	163 (30.24)	0.7564
Q9:Suicidal whish	47 (5.96)	19 (7.56)	28 (5.18)	0.1708
Q10:Crying	186 (23.66)	90 (36.59)	96 (17.78)	<0.0001*
Q11:Agitation	356 (45.12)	117 (47.18)	239 (44.18)	0.4318
Q12:Loss of interest	235 (29.78)	79 (31.85)	156 (28.84)	0.3893
Q13:Indecisiveness	193 (24.49)	76 (30.77)	117 (21.63)	0.0056*
Q14:Worthless	136 (17.24)	52 (20.97)	84 (15.53)	0.0603
Q15:Loss of energy	579 (73.38)	193 (77.82)	386 (71.35)	0.0561
*Q16:Sleep disturbance*	453 (57.41)	156 (62.90)	297 (54.90)	0.0348*
Q17:Irritability	295 (37.44)	100 (40.32)	195 (36.11)	0.2566
*Q18:Appetite disturbance*	313 (39.67)	121 (48.79)	192 (35.49)	0.0004*
Q19:Concentration	330 (41.83)	115 (46.37)	215 (39.74)	0.0797
Q20:Fatigue	565 (71.61)	189 (76.21)	376 (69.50)	0.0523
*Q21:Loss Interest Sex*	323 (41.09)	128 (51.82)	195 (36.18)	<0.0001*

Note: values in parenthesis are percentages and BDI-II symptoms are considered present with a BDI-II score ≥1 in 0–3 scoring system.

*Statistical significant at level of *P* < 0.05.

## References

[1] AHA Statistical fact sheet—populations 2011 Update.

[2] Barth J, Schumacher M, Herrmann-Lingen C (2004). Depression as a risk factor for mortality in patients with coronary heart disease: a meta-analysis. *Psychosomatic Medicine*.

[3] Frasure-Smith N, Lespérance F (2006). Recent evidence linking coronary heart disease and depression. *Canadian Journal of Psychiatry*.

[4] Lespérance F, Frasure-Smith N, Juneau M, Théroux P (2000). Depression and 1-year prognosis in unstable angina. *Archives of Internal Medicine*.

[5] Doering LV, McKinley S, Riegel B (2010). Gender-specific characteristics of individuals with depressive symptoms and coronary heart disease. *Heart and Lung*.

[6] Frasure-Smith N, Lespérance F, Juneau M, Talajic M, Bourassa MG (1999). Gender, depression, and one-year prognosis after myocardial infarction. *Psychosomatic Medicine*.

[7] Weissman MM, Olfson M (1995). Depression in women: implications for health care research. *Science*.

[8] Wolk  SI, Weissman MM, Oldham JM, Riba MB (1995). Women and depression: an update. *Review of Psychiatry*.

[9] Kessler RC (1994). Sex and depression in the National Comorbidity Survey. II: cohort effects. *Journal of Affective Disorders*.

[10] Bland RC, Orn H, Newman SC (1988). Lifetime prevalence of psychiatric disorders in Edmonton. *Acta Psychiatrica Scandinavica*.

[11] Wittchen HU, Ahmoi Essau C, Von Zerssen D, Krieg JC, Zaudig M (1992). Lifetime and six-month prevalence of mental disorders in the Munich Follow-up Study. *European Archives of Psychiatry and Clinical Neuroscience*.

[12] Preisig M, Merikangas KR, Angst J (2001). Clinical significance and comorbidity of subthreshold depression and anxiety in the community. *Acta Psychiatrica Scandinavica*.

[13] Maj M (2005). “Psychiatric comorbidity”: an artefact of current diagnostic systems?. *British Journal of Psychiatry*.

[14] Silverstein B (1999). Gender difference in the prevalence of clinical depression: the role played by depression associated with somatic symptoms. *American Journal of Psychiatry*.

[15] Wenzel A, Steer RA, Beck AT (2005). Are there any gender differences in frequency of self-reported somatic symptoms of depression?. *Journal of Affective Disorders*.

[16] Silverstein B (2002). Gender differences in the prevalence of somatic versus pure depression: a replication. *American Journal of Psychiatry*.

[17] Silverstein B, Caceres J, Perdue L, Cimarolli V (1995). Gender differences in depressive symptomatology: the role played by “anxious somatic depression” associated with gender-related achievement concerns. *Sex Roles*.

[18] Silverstein B, Clauson J, Perdue L, Carpman S, Cimarolli V (1998). The association between female college students’ reports of depression and their perceptions of parental attitudes regarding gender. *Journal of Applied Social Psychology*.

[19] Théroux P, Fuster V (1998). Acute coronary syndromes: unstable angina and non-Q-wave myocardial infarction. *Circulation*.

[20] Thombs BD, Ziegelstein RC, Beck CA, Pilote L (2008). A general factor model for the Beck Depression Inventory-II: validation in a sample of patients hospitalized with acute myocardial infarction. *Journal of Psychosomatic Research*.

[21] Acton GS, Prochaska JJ, Kaplan AS, Small T, Hall SM (2001). Depression and stages of change for smoking in psychiatric outpatients. *Addictive Behaviors*.

[22] Beck CT, Gable RK (2001). Further validation of the postpartum depression screening scale. *Nursing Research*.

[23] Grace SL, Abbey SE, Kapral MK, Fang J, Nolan RP, Stewart DE (2005). Effect of depression on five-year mortality after an acute coronary syndrome. *American Journal of Cardiology*.

[24] Steer RA, Ball R, Ranieri WF, Beck AT (1999). Dimensions of the Beck Depression Inventory-II in clinically depressed outpatients. *Journal of Clinical Psychology*.

